# Effect of Yellow-Tinted Lenses on Visual Attributes Related to Sports Activities

**DOI:** 10.2478/hukin-2013-0003

**Published:** 2013-03-28

**Authors:** Yoshimitsu Kohmura, Shigeki Murakami, Kazuhiro Aoki

**Affiliations:** 1 School of Health and Sports Science, Juntendo University.; 2 Murakami Eye Clinic.

**Keywords:** sports, low-contrast visual acuity, contrast sensitivity, dynamic visual acuity

## Abstract

The purpose of this study was to clarify the effect of colored lenses on visual attributes related to sports activities. The subjects were 24 students (11 females, 13 males; average age 21.0 ±1.2 years) attending a sports university. Lenses of 5 colors were used: colorless, light yellow, dark yellow, light gray, and dark gray. For each lens, measurements were performed in a fixed order: contrast sensitivity, dynamic visual acuity, depth perception, hand-eye coordination and visual acuity and low-contrast visual acuity. The conditions for the measurements of visual acuity and low-contrast visual acuity were in the order of Evening, Evening+Glare, Day, and Day+Glare. There were no significant differences among lenses in dynamic visual acuity and depth perception. For hand-eye coordination, time was significantly shorter with colorless than dark gray lenses. Contrast sensitivity was significantly higher with colorless, light yellow, and light gray lenses than with dark yellow and dark gray lenses. The low-contrast visual acuity test in the Day+Glare condition showed no significant difference among the lenses. In the Evening condition, low-contrast visual acuity was significantly higher with colorless and light yellow lenses than with dark gray lenses, and in the Evening+Glare condition, low-contrast visual acuity was significantly higher with colorless lenses than with the other colors except light yellow. Under early evening conditions and during sports activities, light yellow lenses do not appear to have an adverse effect on visual attributes.

## Introduction

The visual attributes of athletes have attracted attention from researchers in sports science and sports medicine (Stine et al., 1982; Christenson and Winkelstein, 1988; Zwierko, 2007; Zwierko et al., 2010). According to previous reports, athletes tend to have better visual attributes than non-athletes, and competitive athletes tend to have better visual attributes than other athletes. Therefore, visual attributes are important factors for those who participate in sports activities. Typical visual attributes involved in sports activities include: dynamic visual acuity, which is the ability to recognize a moving target; depth perception, which is related to a sense of distance; hand-eye coordination; contrast sensitivity; and low-contrast visual acuity (Hoffman et al., 1984; Hitzeman and Beckerman, 1993; Erickson et al., 2009; Laby et al., 2011).

In general, athletes protect their eyes from ultraviolet rays by wearing sunglasses (Miller, 1974; Fishman, 1986; Cooper et al., 2001). Several previous reports have investigated eye diseases and disorders related to ultraviolet rays and blue light rays which have a short wavelength along the visible light spectrum (Ham et al., 1976; Taylor et al., 1988; Zigman, 1992; McCarty and Taylor, 2002). Lawler et al. (2007) investigated the use of sunglasses by field hockey, soccer, and tennis players. The lens color of conventional sunglasses is usually black, but blackening and darkening athletes’ vision may negatively affect performance in some sports. Therefore, it is difficult to say that black lenses with low luminous transmittance are suitable for all sports. Currently, lenses of various colors and luminous transmittances are available, but it may be difficult for athletes and other consumers to choose the proper lens for certain occasions.

Yellow lenses are one example of a novel type of lens that is currently attracting attention. The color yellow cuts blue light rays, and it gives a high luminous transmittance around a wavelength of 550 nm, to which the human eye is very sensitive. Several authors have already studied the effect of yellow lenses on aspects of visual performance such as contrast sensitivity (Kelly et al., 1984; Yap, 1984; Rieger, 1992; Leguire et al., 1993; Provines et al., 1997; Wolffsohn et al., 2000). However, opinions on this effect are divided, and few of the previous studies selected appropriate measurement items and subjects for sports activities. Moreover, it is likely that product-development research on color lenses examines only the wearer’s comfort rather than the impact of the lenses on visual attributes. It would be very interesting to clarify the effect of yellow lenses on visual performance, which is considered to be an important factor for athletes. It is also necessary to study the effect under various conditions, such as a dim environment or the presence of a glaring light source.

Taking into account various conditions encountered during sports activities, the present study aimed to clarify the effect of various types of color lenses on visual attributes. It is anticipated that effects of lenses color on visual attributes vary among lenses because luminous transmittance differ among color lenses. The results are expected to be of help in developing new color lenses for athletes and in assisting other types of consumers to choose an appropriate color lens.

## Material and Methods

The subjects were 24 students (11 females, 13 males) at a sport-oriented university. Their average age was 21.0 ± 1.2 years. Subjects whose decimal visual acuity, unaided or with contact lenses, was greater than 1.0 (logMAR 0.0) were included; 14 used contact lenses and 10 were unaided. All subjects had sports experience such as Squash, Basketball, Volleyball, Track and field, baseball, tennis, and so on. Contrast sensitivity, dynamic visual acuity, depth perception, and hand-eye coordination were measured with binocular vision. Visual acuity and low-contrast visual acuity were measured with the dominant eye, which was the left eye in 11 subjects and the right eye in 13. The dominant eye was determined by pointing to an object with the index finger or placing the object in a circle made with the hands.

The subjects provided their written, informed consent before participating in the experiment. This study was approved by the Research Ethics Committee of the Juntendo University Graduate School of Health and Sports Science.

**Figure 1:**
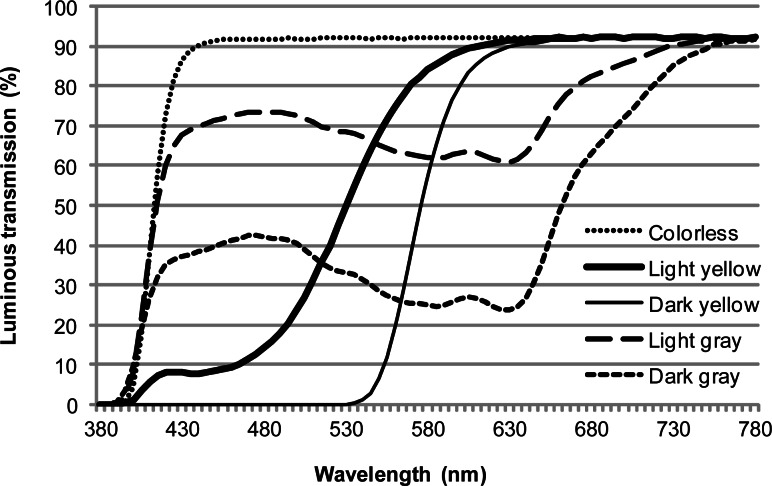
Visible light transmission of the lenses used in this experiment is shown. The luminous transmittance value is 92.0% for colorless lenses, 65.2% for light yellow lenses, 30.4% for dark yellow lenses, 65.9% for light gray lenses, and 30.2% for dark gray lenses

Lenses of 5 colors were used: C, LY, DY, LG, and DG. Figure 1 shows the luminous transmittance of each lens. The five colors of lenses used in this experiment were: colorless (C; luminous transmittance 92.0%), light yellow (LY; luminous transmittance 65.2%), dark yellow (DY; luminous transmittance 30.4%), light gray (LG; luminous transmittance 65.9%), and dark gray (DG; luminous transmittance 30.2%).

### Measurement Procedure

Before measuring, each experimental lens (experimental spectacles) was fitted by an optician for each subject. Lenses of each color were used in random order. For each lens condition, measurements were performed in a fixed order: contrast sensitivity, dynamic visual acuity, depth perception, hand-eye coordination and visual acuity and low-contrast visual acuity. These measurements for each lens were considered a set, and an interval of >15 min was taken between sets. The measurement method is outlined below. A questionnaire was also administered at the end of the experiment.

**Figure 2:**
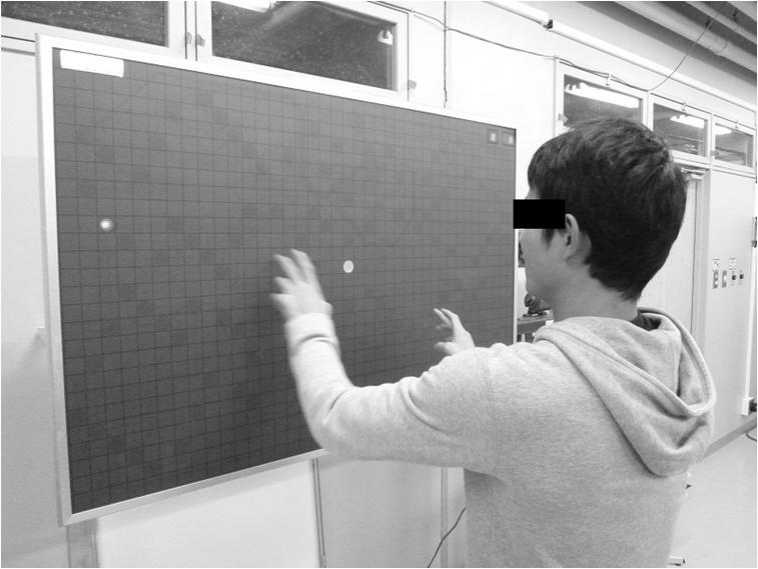
Device for measuring hand-eye coordination.

1. *Visual acuity and low-contrast visual acuity*. Visual acuity and low-contrast visual acuity were measured using a contrast sensitivity acuity tester (CAT-CP, NEITZ Co., Ltd., Tokyo, Japan) (Lee et al., 2001). The subject looked into the tester and attempted to determine the direction of the gap in a Landolt ring. Measurement was performed automatically. The conditions for the measurement were in the order of Evening, Evening+Glare, Day, and Day+Glare. The luminances of the visual target were 200 cd/m^2^ in the Day condition and 10 cd/m^2^ in the Evening condition. The illuminance of Glare with light-emitting diode (LED) light was 200 lx. Under each condition, a visual acuity test (contrast 100%) and low-contrast visual acuity tests (contrast 10% and 5%) were performed. Measurements were performed with the dominant eye in the order of visual acuity, low-contrast visual acuity of 10%, and low-contrast visual acuity of 5%. The visual acuity and low-contrast visual acuity were measured by logMAR values. In the 5% and 10% contrast conditions, if the subject could not determine the gap even for the lowest value of the tester (log MAR 1.3), the data were processed as logMAR 1.4.
2. *Contrast sensitivity*. Contrast sensitivity is ability to distinguish between dark and light. Contrast sensitivity was measured using a Sine Wave Contrast Test (Stereo Optical Co., Inc., Chicago, IL, USA) (Kohmura et al., 2008; Furuta et al., 2009), and contrast sensitivity was measured at each spatial frequency of 1.5, 3, 6, 12, and 18 cycles/degree. Each of the circles in the cart contains lines. The subject attempted to determine the direction of the line (left, right or up). The distance between the subject and the chart was 3.0 m.
3. *Dynamic visual acuity*. Dynamic visual acuity was measured using a dynamic visual acuity test apparatus (HI-10, Kowa Co., Ltd., Aichi, Japan) (Kohmura et al., 2008). In this test, the subject attempted to determine the direction of the gap in a Landolt ring moving from left to right on a semi-circular screen. The rotational speed of the Landolt ring gradually decreased from 49.5 rpm. The subject pressed the switch as soon as he or she determined the direction of the gap in the Landolt ring, and immediately gave an answer. If the answer was correct, the rotational speed when the subject pressed the switch was recorded. The size of the Landolt ring was equivalent to the decimal visual acuity 0.025 (logMAR: 1.6). The Landolt ring was projected by a slide projector onto a 120 cm semicircular screen. The screen was located 80 cm away from the subject. The luminance of this visual target was about 1300 cd/m^2^. The direction of the gap of the Landolt ring could be up, down, left, or right, and it was presented in random order. The measurement was repeated until five records were obtained, and the average of the records was considered the measured value. If the subject made three or more mistakes, the test was re-started.
4. *Depth perception*. Depth perception was measured using a depth perception test apparatus (AS-7JS1, Kowa Co., Ltd.) (Kohmura et al., 2008; Furuta et al., 2009). The apparatus contained three bars, and the central bar moved back and forth at a speed of 50 mm/s. The other two bars on the sides were fixed. Subjects were able to see the bars from a window in the device. The subject attempted to press a switch to stop the central bar when he or she felt that the three bars were laterally positioned in line with each other. After two practice runs, three measurements were performed. The absolute value of the displacement between the central bar and the other two bars was recorded. This displacement was measured in millimeters. The distance between the subject and the apparatus was 2.5 m.
5. *Hand-eye coordination*. Hand-eye coordination was measured using the AS-24 (Kowa Co., Ltd.) (Wada et al., 2007; Uematsu et al., 2009) (Figure 2). The test apparatus had 120 lamps on its panel, and the lamps were lit one at a time in random order. After a lamp was lit, the next lamp was lit 1.3 sec later, or immediately if the subject pushed the lamp. The subject attempted to push the lamps as accurately and quickly as possible, and the time (seconds) taken to light the 120 lamps was recorded. One measurement was performed for each lens.
6. *Questionnaire*. Using visual analog scales, each question was answered by marking on a 100-mm line (the length in millimeters was not written on the questionnaire sheet) on the basis of a subjective assessment and impression. The questionnaire was conducted after all the visual attribute measurements were finished. The positions of the marks made by the subjects were measured in millimeters. Subjects were asked to evaluate the following five qualities: brightness (Bright: 100 mm, Dark: 0 mm), sharpness (Sharp: 100, Blurry: 0), changes in color recognition (Not changed: 100, Changed: 0), glare (when looking at a fluorescent light in the room) (No glare: 100, Glare: 0), and overall impression (Good: 100, Bad: 0).

*Statistical Processing*. Two-way repeated measures analysis of variance (ANOVA) with factors of contrast and lens was used for contrast sensitivity and low-contrast visual acuity. Oneway repeated measures ANOVA was used for dynamic visual acuity, depth perception, hand-eye coordination, visual acuity, and the questionnaire. The significance level was set at p< 0.05.

## Results

Measurement Results of Visual Attributes are presented in Table 1.

For low-contrast visual acuity, in every condition, the interaction was not significant and the main effect of contrast was significant at p<0.01 (Day: F=98.10, p=0.00, Day+Glare: F=215.55, p=0.00, Evening: F=199.63, p=0.00, Evening+Glare: F=160.73, p=0.00).

In the Day condition, the main effect of lens was significant at p<0.05 (F=2.68, p=0.04), and multiple comparisons showed a significant difference between C and DG at p<0.05 (p=0.04). In the Day+Glare condition, there was no significant difference. In the Evening condition, the main effect of lens was significant at p< 0.01 (F=4.80, p=0.00), and C and LY had significant differences from DG at p<0.01 (C vs. DG: p=0.00, LY vs. DG: p=0.01). Finally, in the Evening+Glare condition, the main effect of lens was significant at p<0.01 (F=5.37, p=0.00), and C had significant differences from DY (p=0.02), LG (p=0.01), and DG (p=0.00).

For contrast sensitivity, the interaction was not significant, and the main effects of lens and contrast were significant at p<0.01 (lens: F=10.54, p=0.00, contrast: F=178.34, p=0.00).

DY and DG had significant differences from C, LY, and LG. C vs. DY (p=0.01), LY vs. DY (p=0.00), LG vs. DY (p=0.00), and LG vs. DG (p=0.00) were significant at p<0.01. C vs. DG (p=0.01) and LY vs. DG (p=0.02) were significant at p<0.05.

For dynamic visual acuity and depth perception, there were no significant differences. For hand-eye coordination, the result of ANOVA was significant at p<0.05 (F=3.48, p=0.01), and the result of the multiple comparisons showed a significant difference between C and DG at p<0.05 (p=0.05).

For visual acuity, there were no significant differences in the Day, Day+Glare, and Evening conditions. However, in the Evening+Glare condition, the result of ANOVA was significant at p<0.05 (F=3.48, p=0.01).

Questionnaire results are presented in Table 2.

For all items included in the questionnaire, the result of one-way ANOVA was significant at p<0.01 (brightness: F=27.34, p=0.00, sharpness: F=9.63, p=0.00, color: F=71.15, p=0.00, glare: F=7.90, p=0.00, overall: F=16.14, p=0.00). The results of the multiple comparisons were as follows: regarding the question about brightness, C presented significant differences from LG (p=0.00) and DG (p=0.00), LY from DY (p=0.00), LG (p=0.00), and DG (p=0.00), DY from DG (p=0.00), and LG from DG (p=0.00); as far as sharpness was concerned, C was significantly different from DY (p=0.01), LG (p=0.03), and DG (p=0.01), LY from DY (p=0.00) and DG (p=0.01), and LG from DG (p=0.01); for the question about changes in color recognition, every combination had a significant difference at p<0.01 (p=0.00), except for LY and DY (p=0.01), and LY and DG (p=0.04), where the significance was at p<0.05; for the question about glare, C showed significant differences from LG (p=0.00) and DG (p=0.00), LY from DG (p=0.01), and LG from DG (p=0.04); and with regard to the overall impression, C was significantly different from DY (p=0.00) and DG (p=0.03), LY from DY (p=0.00), DY from LG (p=0.00), and LG from DG (p=0.00).

## Discussion

There were no significant differences among the tested lenses with respect to dynamic visual acuity or depth perception. When recognizing a target in the fovea as in the dynamic visual acuity test, or determining the distances of the three bars by steadily looking at them, the effects of lens color and luminous transmittance are assumed to be minor. In such circumstances, the differences among the lenses used in this experiment appeared to be small. During sports activities, adverse effects of the lenses used in this study on the visual attributes related to tracking a ball or person with the eye or recognizing the positional relationship of a ball and a person might be small.

On the other hand, for hand-eye coordination, the time was significantly shorter for C than DG. Taking into consideration that this time is the time taken to push all the 120 lamps in this measurement, a shorter time represents a better result, where the subject could push the lamps quickly and accurately. In repeatedly finding a lamp in the peripheral vision and pushing it accurately, DG appears to have some effects. In sports that require quick and accurate responses, using DG lenses may have some adverse effects. It is suggested that the extent of this effect could be different between situations in which the eyes are always directed to the target and those in which a response to a target in the peripheral vision is required.

Contrast sensitivity was significantly higher for C, LY, and LG than for DY and DG, and in the Day condition, low-contrast visual acuity was significantly higher for C than DG. Therefore, it is assumed that a low luminous transmittance may affect contrast-related features of the lens under normal circumstances such as daylight. However, the low-contrast visual acuity test in the Day+Glare condition showed no significant difference among the lenses. In a glare situation, such as in the presence of glare during daylight, all lenses tested in this study appeared to have little effect on the contrast attributes. Therefore, in a bright situation during daylight, using a low luminous transmittance lens might not significantly affect visual attributes. On a sunny day, a lens with low luminous transmittance is assumed to be usable without an effect on low-contrast visual acuity. Recently, although the usage and situations were very different from the lenses used in this study, there have also been reports regarding the effect of colored contact lenses in sports activities (Porisch, 2007; Cerviño et al., 2008). Erickson et al. (2009) studied and reported the effect of colored contact lenses; they found that using amber and gray-green contact lenses with luminous transmittances of 50% and 36%, respectively, could achieve better contrast sensitivity than using colorless lenses in bright sunlight.

In contrast to the above discussed Day conditions, in the Evening condition, low-contrast visual acuity was significantly higher for C and LY than DG, and in the Evening+Glare condition, low-contrast visual acuity was significantly higher for C than for other colors except LY. Therefore, particularly under slightly dim circumstances such as early evening, LY lenses have little effect on low-contrast visual acuity and may be usable in the same manner as colorless lenses. That is to say, it is expected that even in a dim environment where a single strong light source is present, such as sunset, LY lenses can prevent injuries and make the vision brighter while maintaining the visual attributes.

For the question about brightness, C and LY were rated light, DG was rated dark, and DY and LG were considered the same. As for sharpness, C was rated sharper than any lenses other than LY. For each color, the light lenses seemed to be considered sharper than the dark lenses, and LY was rated sharper than DG. It has been previously reported that yellow lenses seem brighter (Kelly, 1990; Chung and Pease, 1999), and this could be said to be a major feature of yellow lenses.

As for changes in color recognition, C was rated to cause the least change, followed by LG, DG, LY, and DY. The subjects seemed to have felt that yellow lenses affected their color recognition, as reported in previous studies (Hovis et al., 1989; de Fez et al., 2002). Several advantages of yellow lenses have been reported, as mentioned above, but the drawback of impaired color recognition must be taken into consideration. As for glare, DG was rated to have less glare than C, LY, and LG, and LG was rated to have less glare than C. Finally, as for the overall impression, C and LG were rated better than the dark lenses, and LY was rated better than DY.

Based on the above presented results, other than the question about glare, ratings for dark colors tended to be lower than those for the light colors. Generally speaking, people are most accustomed to using black lenses, and yellow lenses are unfamiliar because they change the wearer’s color recognition. To encourage consumers to use yellow lenses, it is important to inform them of both the advantages and disadvantages. Yellow lenses can be used during sports activities in which color recognition is not critical. However, in sports where many athletes participate with various colored uniforms, changes in color recognition may have adverse effects on performance, and colored lenses must therefore be used with care. Recently, the effects of lenses of other colors that were not used in this study have also been studied (Lee et al., 2002). In the future, it will be necessary to conduct studies investigating which lenses are suited for different practical situations, including sports activities.

Dark lenses appear to have a greater effect on contrast-related features, whereas under early evening conditions, LY could be used without adverse effects on visual attributes. Under bright conditions, such as in the daylight with glare, the lenses used in this study do not appear to be significantly different in their measured effects on visual attributes.

Moreover, when tracking the position of or determining the distance of a target by looking steadily at the target, the effects of the studied lenses on visual attributes appear to be small. However, when it is necessary to locate a target in the peripheral vision and quickly and accurately respond, as in ball sports, DG may have adverse effects. Therefore, depending on the type of sport, using dark colored lenses may affect athletes’ performance.

According to the subjective opinions collected through the questionnaire, LG, a light and familiar black color, appeared to be better rated than the other lenses. This may have been because the subjects were used to black lenses and were uncomfortable with yellow lenses because they changed their color recognition and the brightness of the field of view.

The results of this experiment show that under early evening conditions and during sports activities, light yellow lenses do not appear to have an adverse effect on visual attributes. It is assumed that using yellow-tinted lenses during sports activities is one of the useful methods to protect eyes from ultraviolet rays and a blue light without adverse effect on visual attributes. However, color lenses need to be developed further, taking into account how the color of the lens and the luminous transmittance properties affect the visual attributes and subjective feelings of the wearer. Furthermore, the subjects of this study were young, with an average age of 21 years; taking into account that visual attributes change with age, it is also important to study the effects of colored lenses on visual attributes in subjects in other age groups.

## Figures and Tables

**Figure 3:**
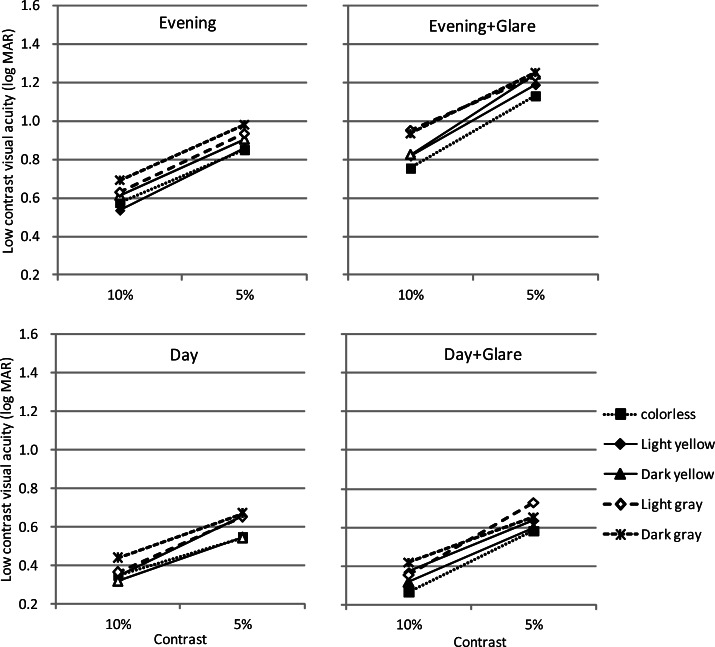
Measurements results for low-contrast visual acuity are shown. In the Day condition, the statistical analysis shows a significant difference between colorless and dark gray at p<0.05. In the Day+Glare condition, there is no significant difference. In the Evening condition, colorless and light yellow have significant differences from dark gray at p<0.01. In the Evening+Glare condition, colorless is significantly different from dark yellow (p<0.05), light gray (p<0.01), and dark gray (p<0.01).

**Figure 4:**
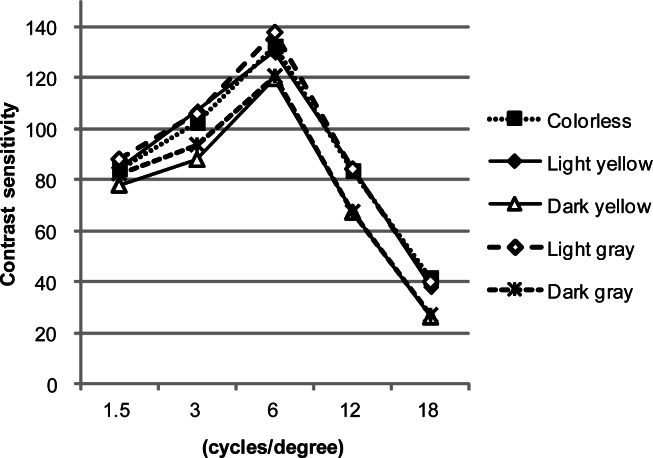
Means of contrast sensitivity with each of the lenses are shown. Dark yellow and dark gray have significant differences from colorless, light yellow and light gray. Colorless vs. dark yellow, light yellow vs. dark yellow, light gray vs. dark yellow, and light gray vs. dark gray are significantly different at p<0.01. Colorless vs. dark gray and light yellow vs. dark gray are significantly different at p<0.05.

**Table 1:** Mean and standard deviation of visual performance related to sports activities with each of the lenses.

		Dynamic visual acuity	Depth perception	Eye-hand coordination	Visual acuity (log MAR)			

		(rpm)	(mm)	(sec)	Evening	Evening +Glare	Day	Day +Glare
Colorless	M	43.4	8.4	87.7	0.07	0.06	−0.01	−0.02
	SD	3.2	5.1	7.0	0.18	0.19	0.16	0.13
Light yellow	M	42.7	9.1	88.3	0.05	0.05	−0.02	−0.05
	SD	3.8	5.6	6.2	0.20	0.17	0.14	0.09
Dark yellow	M	42.4	6.9	88.6	0.09	0.09	−0.04	−0.06
	SD	3.3	7.6	6.1	0.17	0.19	0.17	0.06
Light gray	M	43.4	8.9	88.0	0.09	0.15	−0.01	0.01
	SD	3.2	7.0	6.7	0.22	0.23	0.18	0.21
Dark gray	M	43.0	10.9	90.3	0.09	0.14	−0.03	−0.02
	SD	3.3	12.3	6.5	0.14	0.19	0.12	0.17

**Table 2:** Questionnaire results

Score	Brightness		Sharpness		Color		Glare		Overall	

	M	SD	M	SD	M	SD	M	SD	M	SD
Colorless	75.2	18.9	77.9	19.5	93.8	11.7	33.2	26.5	77.6	22.2
Light yellow	81.4	12.8	75.7	17.2	33.3	23.1	40.0	25.8	59.8	19.4
Dark yellow	56.8	25.7	52.2	23.6	17.7	18.1	51.1	27.7	32.7	22.3
Light gray	50.4	22.5	61.7	21.8	73.3	20.5	54.2	28.6	73.0	17.2
Dark gray	28.2	23.4	53.2	26.4	56.2	27.3	69.3	26.7	50.0	27.3
